# Occurrences of Indigestible Foreign Bodies in Cattle Slaughtered at Morogoro Municipal Slaughterhouse, Tanzania

**DOI:** 10.1155/2018/4818203

**Published:** 2018-02-22

**Authors:** S. F. Bwatota, M. Makungu, H. E. Nonga

**Affiliations:** ^1^Department of Veterinary Surgery and Theriogenology, College of Veterinary Medicine and Biomedical Sciences, Sokoine University of Agriculture, P.O. Box 3020, Morogoro, Tanzania; ^2^Department of Veterinary Medicine and Public Health, College of Veterinary Medicine and Biomedical Sciences, Sokoine University of Agriculture, P.O. Box 3021, Morogoro, Tanzania

## Abstract

A cross-sectional study was conducted to determine the occurrence of indigestible foreign bodies (IFB) in cattle slaughtered at Morogoro Municipal Slaughterhouse, Tanzania. A total of 387 slaughter cattle were examined for presence of IFB. Out of 387 examined cattle, 93 (24.03%) had IFB in their forestomachs. The observed IFB were plastic bags, fruit seeds, clothing materials, ropes, hairballs, leather materials, stones, metallic nails, and wire. Plastic bags were the most frequently (50.5%) observed IFB followed by fruit seeds (18.3%). A significantly (*p* < 0.05) high proportion of old animals (31.7%) had IFB compared to the young animals (21.2%). Similarly, the frequency of occurrence of IFB was significantly high (*p* < 0.05) in crossbred dairy cattle (42.3%) compared to local breeds (22.7%). Cattle that appeared with poor body condition (37.8%) were found to be more affected (*p* < 0.05) by IFB than those with good body condition (15.9%). In 91.4% of animals which had IFB, all the materials were located in the rumen. This study showed that presence of IFB is a common problem in cattle slaughtered at Morogoro Municipal Slaughterhouse and may significantly cause poor production and mortality in affected animals. Therefore, appropriate solid waste disposal should be implemented.

## 1. Introduction

Tanzania has the third largest cattle population in Africa with 25 million heads of cattle, of which 98% are indigenous breed which are extensively managed by small scale traditional farmers [1]. Beef cattle accounts for more than 50% of the meat produced in the country [2]. Despite the presence of a large number of populations of heads of cattle in the country, the livestock sector contributes only 7.4% to the country's Gross Domestic Product [1]. Nevertheless, cattle production in Tanzania plays an important role in national food supply and food security. Furthermore it acts as a source of employment, energy, cash income, and a living saving bank in periods of crop failure and economic distress. Additionally, it provides manure and draught animal power, therefore contributing to sustainable agriculture [1, 3]. However, the livestock industry is constrained by various factors such as inadequate technical support services, infrastructure, marketing system, diseases, and low genetic potential [3, 4].

Environmental contamination with solid wastes from domestic and commercial sources is common in developing countries like Tanzania because of a poor refuse disposal system. The solid wastes may range from metallic to nonmetallic type and, of recent, plastic bags are common in various periurban and urban areas of Tanzania. Unfortunately, cattle commonly ingest the indigestible foreign bodies (IFB) as a result of their indiscriminate feeding habits [5, 6]. The IFB may go and lodge to different parts of the gastrointestinal tract especially in the forestomachs. Presence of IFB, apart from causing pathological lesions, reduces the volume of the forestomachs which is otherwise supposed to be filled with feed. The occupation of space by IFB ultimately leads to reduced feed intake. Consequences to these are economic losses due to severe loss of production and increased mortality rates [7, 8]. Several authors have associated the ingestion of IFB in cattle with industrialization, agriculture mechanization, food scarcity, poor farming management, and diseases that cause pica [5–7, 9, 10].

Cases of occurrence of IFB in forestomachs of cattle have been reported in Tanzania [8] and in other African countries such as Rwanda [5], Ethiopia [11], Nigeria [12], and Ghana [13]. Metallic and nonmetallic foreign bodies that were isolated included nails, plastic bags, sewing needles, clothes, pieces of milling machine sieves, and hairballs [5, 8]. Morogoro region is among the areas in Tanzania with the high number of cattle that are extensively managed under the traditional system. Kilosa, Mvomero, and Gairo are the districts with the cattle population that sums to 400,000 where most of the slaughter cattle at Morogoro Municipal Slaughterhouse are sourced there [14]. The aim of this study was to investigate the occurrences of indigestible foreign bodies in cattle slaughtered at Morogoro Municipal Slaughterhouse so as to determine the magnitude of the condition for appropriate control measures.

## 2. Materials and Methods

### 2.1. Study Area and Design

This cross-sectional study was conducted in September 2017 at the Morogoro Municipal Slaughterhouse, in Morogoro region. The Morogoro Municipality is about 200 km west of Dar es Salaam [15] and lies between latitudes 5°7′ and 10°00′ south of the Equator and longitudes 35°6′ and 39°5′ east of Greenwich [16]. On average, 100–120 cattle are slaughtered per day at Morogoro Municipal Slaughterhouse. The slaughter cattle originate mostly from different areas of Morogoro region in particular Kilosa, Mvomero, and Gairo.

### 2.2. Sample Size and Selection of Study Animals

A total of 387 cattle slaughtered at Morogoro Municipal Slaughterhouse were examined during the one-month visit at the slaughterhouse. The sample size was calculated based on the formula *n* = 1.96^2^*p*(1 − *p*)/*L*^2^ by Thrustfied [17], where *n* is required sample size, *p* is prevalence of IFB, and *L* is precision. Since the prevalence of IFB was unknown, a prevalence of 50% was estimated at 95% confidence and a precision of 5% was used.

During the visit at the slaughterhouse, study animals were being selected from the crash when they were aligned to the slaughter hall using systematic random sampling at an interval of every five animals. Therefore, every fifth slaughtered animal was selected for inspection of IFB in the stomachs during postmortem examination.

For all the animals that were selected for study, the biodata like breed, age, sex, body condition score, grazing system, and the source of the animal was recorded. The age of the animal was determined by dentition as previously described by Pace and Wakeman [18]. Selected animals were categorized as ≤4 years or >4 years. The body condition was recorded as poor, moderate, and good based on appearance of the animal and manual palpation of the dorsal spines and transverse processes of the lumbar vertebrae [19]. The grazing system and the source of the animals were explored from the owners.

### 2.3. Postmortem Inspection for Indigestible Foreign Bodies

After the cattle was slaughtered and eviscerated, the rumen, reticulum, omasum, and abomasum were put aside for detailed examination of IFB. The set of stomachs was put on clean plastic sheet spread on the floor and straightened to clearly display the rumen, reticulum, omasum, and abomasum. Each stomach part was transversely cut to separate and then longitudinally sliced and the contents were being carefully emptied while inspecting for any material which is not of animal feed and was being classified as IFB. When the materials were unclearly identified, they were being washed with tap water for clear visibility. The encountered IFB were recorded for each site.

### 2.4. Data Analysis

Data collected was entered into Microsoft Excel spreadsheet (Microsoft 2010) and was analyzed using Epi Info™ version 7.2.2.2 (Centers for Disease Control and Prevention, Atlanta, 2017). Frequencies (percentages) for categorical variables were calculated and chi-square test was used for comparison. Statistically significant difference was accepted at a probability (*p*) of *p* < 0.05.

## 3. Results

### 3.1. General Information of Study Animals

A total of 387 animals were examined for presence of IFB at the Morogoro Municipal Slaughterhouse. The greater proportion (73.9%) of the cattle that were examined originated from the Nanenane/Tungi cattle auction market. The rest of the animals (26.1%) originated from different areas in Morogoro region such as Dakawa, Mkongeni, Kitungwa, Kilosa, Kilakala, and Kichangani. Up to 93.3% of the examined cattle were local breeds, specifically Tanzanian short horn zebu (TSZ) (80.1%), Boran (18.8%), and Ankole (1.1%). Only 6.7% were dairy crossbred cattle (Table 1). Most of the examined animals (56.1%) were females (Table 1).

### 3.2. Occurrences of IFB in Slaughtered Cattle

Out of 387 examined cattle, 93 (24.03%) had different kinds of IFB in different forestomachs. The frequency of occurrence of IFB was significantly high (*p* < 0.05) in intensively managed compared to extensively managed cattle (Table 1). From the 361 local breeds and 26 crossbred dairy cattle examined, it was observed that the proportion of crossbred dairy cattle with IFB was significantly (*p* < 0.05) high compared to local breeds (Table 1). Out of 387 animals, 73.1% were less than or equal to four years old, whereas 26.9% were more than four years old. There was statistically significant difference (*p* < 0.05) in the frequency of occurrence of IFB among the two age groups (Table 1). Among the different body condition categories observed, the highest frequency (37.8%) of occurrence of IFB was recorded in animals with poor body condition. Statistically significant difference was observed between animals with poor body condition and those with good body condition (Table 1).

**Figure 1:**
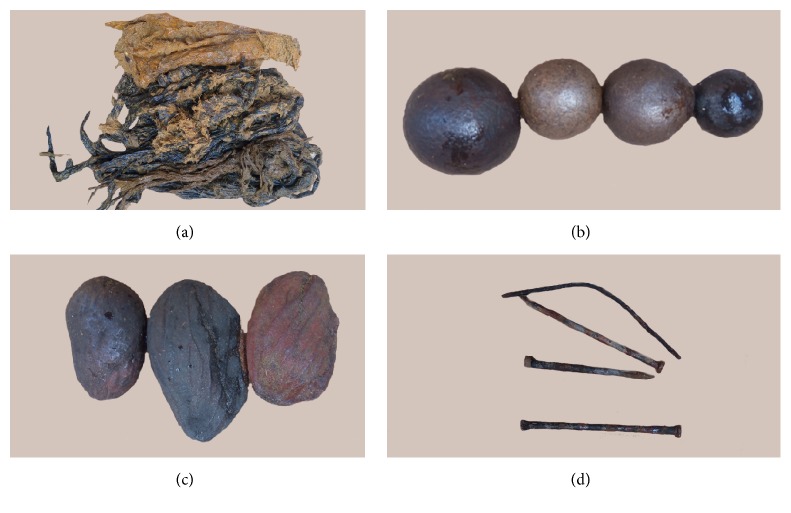
Different types of indigestible foreign bodies in cattle slaughtered at Morogoro Municipal Slaughterhouse, Tanzania. (a) Plastic bags; (b) fruit seeds from avocado; (c) fruit seeds from mango; (d) metallic wire and nails.

**Figure 2:**
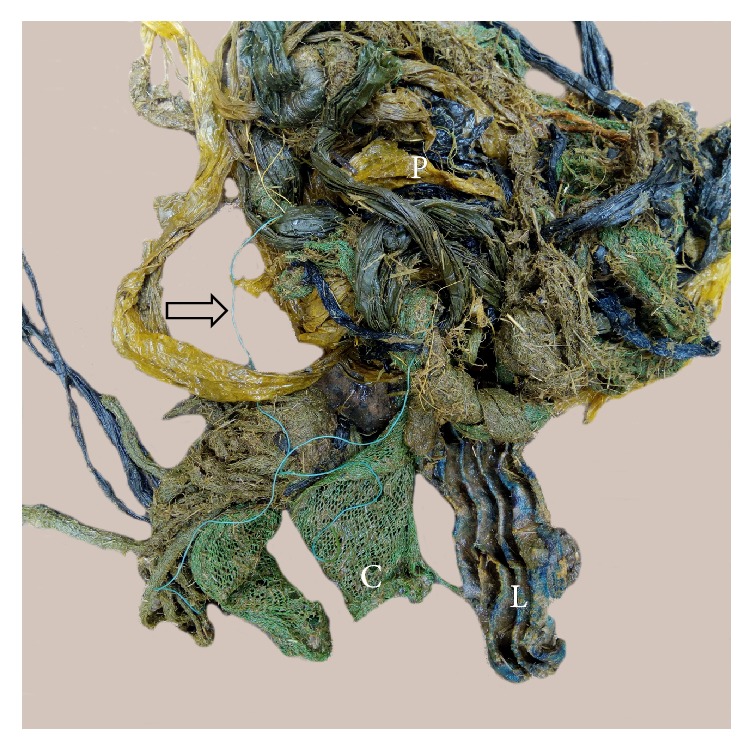
A mix of indigestible foreign bodies from a cow with a poor body condition slaughtered at Morogoro Municipal Slaughterhouse. P = plastic bags; L = leather; C = clothing material. A rope is indicated by a black open arrow.

**Figure 3:**
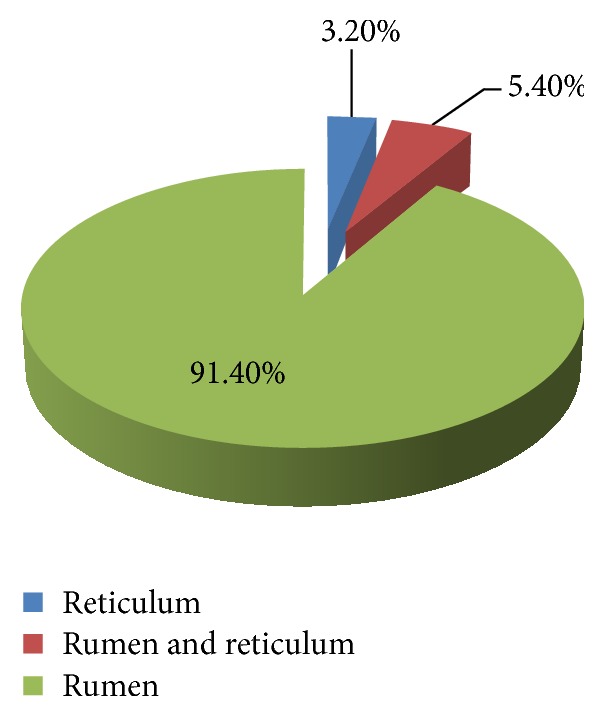
The distribution of indigestible foreign bodies in different compartments in cattle slaughtered at Morogoro Municipal Slaughterhouse, Tanzania.

Different types of IFB were observed which included plastic bags, fruit seeds, clothing materials, ropes, hairballs, leather materials, stones, metallic nails, and wire (Figure 1). Among the different kinds of IFB encountered, plastic bags were the most frequently observed (50.5%) followed by fruit seeds (18.3%) from avocados and mangoes (Table 2). A mix of IFB were seen in eight animals (Figure 2). In up to 91.4% of the total animals with IFB, the foreign materials were present in the rumen (Figure 3). The metallic nails and wire (Figure 1(d)) were seen in the reticulum. None of the IFB was seen in the omasum and abomasum.

## 4. Discussion

The purpose of this study was to assess the occurrences of IFB in cattle slaughtered at Morogoro Municipal Slaughterhouse, Tanzania. It was found that 24.03% of the slaughtered cattle had IFB in the forestomachs. The occurrence rate of IFB in cattle obtained during this study is higher than the previously reported studies in Rwanda [5] and Nigeria [12] which was 17.4% and 12%, respectively. However, it was lower than that reported in Ethiopia [19] and Pakistan [20] which had a prevalence of 43.4% and 59.14%, respectively. The variation in the prevalence of IFB in the reported areas may be the result of different levels of environmental contamination with IFB. Higher prevalences have been associated with high levels of environmental contamination with foreign bodies especially in urban areas [21]. Additionally, this may be due to seasonal variation in which the studies were conducted. The occurrence of IFB has been reported to be high in dry season where there is scarcity of forage, which makes the animal eat anything in its immediate surroundings that may include IFB [22]. Further, management system may play a role in the variation of the occurrence of IFB. Lack of supplementation of mineral and vitamins in higher producing animals such as dairy cattle may result in pica which may predispose to consumption of IFB.

The higher prevalence of IFB in crossbred dairy cattle compared to local breeds which was observed in this study may be associated with the level of productivity. The crossbred dairy cattle have got higher productivity than local breed cattle; therefore lack or inadequate supplementation of minerals and vitamins predisposes them more to ingestion of IFB. Moreover, the source/origin of the slaughter crossbred dairy cattle may also play a role. In urban and periurban areas such as Morogoro Municipality, there is a high risk of environmental contamination with IFB, especially plastic bags which are more used but are poorly disposed of. Due to the scarcity of grazing land in urban and periurban areas, the crossbred dairy cattle are normally kept at the backyard and graze very close to households. As such, they are more exposed to ingestion of IFB. The local breed cattle normally originates from rural areas where there is extensive grazing land which is less contaminated with IFB. Additionally, the crossbred dairy cattle are mainly kept for milk production such that once the production is low such animals are culled and they can be sent for slaughter. It is most likely that the majority of female crossbred dairy cattle in this study were sent for slaughter due to low production/productivity as a result of ingestion of IFB. This is supported by the fact that 61.5% of female crossbred cattle were positive for IFB against only 23.0% of male crossbred cattle which had IFB. Previous studies [23–25] have also reported the higher occurrences of IFB in crossbred cattle compared to those in local breeds.

The higher prevalence of IFB in animals more than four years of age when compared to animals less than or equal to four years of age which was obtained in this study may be associated with prolonged exposure to the contaminated environment. Previous studies in Ethiopia [11, 21, 23, 24] and Rwanda [5] have also reported higher prevalence of IFB in older cattle than in young cattle.

In this study, it was also found that animals with poor body condition were more likely to have IFB than those with good body condition. Once ingested, the IFB in the gastrointestinal tract may interfere with the flow of ingesta and absorption of volatile fatty acids (VFA) and hence reduce weight gain [10, 12, 23]. Moreover, IFB may cause anorexia, pain, and fever and hence decreased production and loss of body condition [9, 21, 26]. However, other factors may have contributed to the poor body condition of the animals such as inadequate feeding, old age, and chronic diseases. The higher prevalence of IFB in animals with poor body condition when compared to animals with good body condition has also been reported by several authors [5, 19, 23, 27].

Plastic bags were the most frequently encountered IFB in this study. This finding is in agreement with previous studies in Ethiopia [11, 19, 23, 27] and Rwanda [5]. Plastic bags are commonly used for packaging of different items and in the absence of better means of disposal; they are just thrown on land and scatter all over it and persist in the environment for life since they are nonbiodegradable materials. When cattle are grazed in such contaminated environment, chances of picking the IFB are high as was observed in the current study.

The nonmetallic foreign bodies such as plastic bags, fruit seeds, ropes, clothing, and leather materials which were encountered in this study were thrown as garbage from households. Plastic bags are commonly used for packaging of different items in Morogoro Municipality. Additionally, mangoes and avocados are fruits which are frequently consumed in the municipality households. The metallic foreign bodies such as nails and wires originate mainly from fencing and motor vehicle radial tire wires, milling machines, and farms when repairs are made to yards and fences and in the vicinity of feed trough [8, 28]. The crossbred dairy cattle in Morogoro Municipality are commonly kept at the backyard, being regularly fed with maize bran from local milling machines and grazing close to households. It is most likely that the sources of metallic nails and wire in this study were from milling machines and repairs made to households and backyard structures. Hairballs resulted from ingestion of hair as a result of excessively licking themselves or persistent sucking of pen mates [28, 29]. Excessive licking may be due to a skin disease characterized by itching such as pediculosis or scabies [29]. In cattle, swallowed hairs are formed into oval bodies as a result of churning and rolling movements of the rumen once ingested [29]. Hairballs may cause choke during regurgitation of the cud in adult cattle or obstruction of the pylorus and small intestines in calves [29].

The occurrence of IFB in cattle at Morogoro Municipality indicates that there is a widespread use of plastic bags. Due to a poor disposal system of solid wastes, it may contribute to a significance loss in livestock production. Therefore, the public should be encouraged to use biodegradable materials for packaging of different items.

The higher frequency of occurrence of IFB in the rumen is probably due to its larger volume compared to other compartments, and almost all ingested feed especially of low density settles in the rumen [5, 23, 27]. The metallic foreign bodies, because of their high density, usually localize in the reticulum [7] as was observed in this study.

## 5. Conclusions

It is concluded that ingestion of IFB is common in cattle slaughtered at Morogoro Municipal Slaughterhouse. It may be a significant cause of losses in livestock production. Therefore, appropriate solid waste disposal should be implemented. Although slaughter facilities surveys of livestock health have limitations, they are an economical way of gathering health information since they give the true picture on what is happening in the livestock population.

## Figures and Tables

**Table 1:** Occurrence of indigestible foreign bodies in different categories of cattle slaughtered at Morogoro Municipal Slaughterhouse (*n* = 387).

Parameter	Category	Animals examined	Animals with indigestible foreign bodies	Percent	Odds ratio	95% CI	*p* value
Grazing system	Extensive	361	82	22.7	2.4951	1.1033–5.6429	0.0175^*∗*^
	Intensive	26	11	42.3			

Breed	Cross	26	11	42.3	0.4008	0.1772–0.9064	0.0175^*∗*^
	Local	361	82	22.7			

Sex	Female	217	52	23.9	1.0085	0.6305–1.6131	0.4850
	Male	170	41	24.1			

Age	≤4 years	283	60	21.2	1.7275	1.0459–2.8532	0.0179^*∗*^
	>4 years	104	33	31.7			

Body condition	Poor	37	14	37.8	3.2095	1.2718–8.0995	0.0076^*∗*^
	Good	69	11	15.9			
	Moderate	281	68	24.2	1.6833	0.8359–3.3898	0.0702
	Good	69	11	15.9			

^*∗*^Statistically significant.

**Table 2:** Frequency of occurrence of different types of indigestible foreign bodies in cattle slaughtered at Morogoro Municipal Slaughterhouse.

Type of foreign body	Number (%) of animals with foreign body
Clothing materials	3 (3.2)
Hairballs	3 (3.2)
Leather materials	5 (5.4)
Metallic wire and nails	1 (1.1)
Fruit seeds	17 (18.3)
Plastic bags	47 (50.5)
Ropes	8 (8.6)
Stones	1 (1.1)
Mixed	8 (8.6)
